# Insight into the Possible Formation Mechanism of the Intersex Phenotype of Lanzhou Fat-Tailed Sheep Using Whole-Genome Resequencing

**DOI:** 10.3390/ani10060944

**Published:** 2020-05-29

**Authors:** Jie Li, Han Xu, Xinfeng Liu, Hongwei Xu, Yong Cai, Xianyong Lan

**Affiliations:** 1 Animal Genome and Gene Function Laboratory, College of Animal Science and Technology, Northwest A&F University, Yangling 712100, China; lijie95@nwafu.edu.cn (J.L.); xuhan23@mail2.sysu.edu.cn (H.X.); liuxinfen227@nwafu.edu.cn (X.L.); 2 School of Medicine, Sun Yat-sen University, Guangzhou 510275, China; 3 College of Life Science and Engineering, Northwest Minzu University, Lanzhou 730030, China; 4 Gansu Tech Innovation Center of Animal Cell, Biomedical Research Center, Northwest Minzu University, Lanzhou 730030, China; 5 Science Experimental Center, Northwest Minzu University, Lanzhou 730030, China; caiyong@xbmu.edu.cn; 6 Key Laboratory of Biotechnology and Bioengineering of State Ethnic Affairs Commission, Biomedical Research Center, Northwest Minzu University, Lanzhou 730030, China

**Keywords:** sheep, intersex, whole-genome resequencing, copy number variation, forming mechanism

## Abstract

**Simple Summary:**

Individuals with hermaphroditism are a serious hazard to animal husbandry and production due to their abnormal fertility. The molecular mechanism of sheep intersex formation was unclear. This study was the first to locate the homologous sequence of the goat polled intersex syndrome (PIS) region in sheep and found that the intersex traits of Lanzhou fat-tailed sheep were not caused by the lack of this region. By detecting the selective sweep regions, vital genes associated with androgen biosynthesis and the follicle stimulating hormone response entry were found, including *steroid 5 alpha-reductase 2* (*SRD5A2*), and *pro-apoptotic WT1 regulator (PAWR*). Additionally, the copy number variations of the four regions on chr9, chr1, chr4, and chr16 may affect the expression of the gonadal development genes, *zinc finger protein*, *FOG family member 2* (*ZFPM2*), *LIM homeobox 8* (*LHX8*), *inner mitochondrial membrane peptidase subunit 2* (*IMMP2L*) and *slit guidance ligand 3* (*SLIT3*), respectively, and further affect the formation of intersex traits.

**Abstract:**

Intersex, also known as hermaphroditism, is a serious hazard to animal husbandry and production. The mechanism of ovine intersex formation is not clear. Therefore, genome-wide resequencing on the only two intersex and two normal Lanzhou fat-tailed (LFT) sheep, an excellent but endangered Chinese indigenous sheep breed, was performed. Herein, the deletion of homologous sequences of the goat polled intersex syndrome (PIS) region (8787 bp, 247747059–247755846) on chromosome 1 of the LFT sheep was not the cause of the ovine intersex trait. By detecting the selective sweep regions, we found that the genes related to androgen biosynthesis and follicle stimulating hormone response items, such as *steroid 5 alpha-reductase 2* (*SRD5A2*), *steroid 5 alpha-reductase 3* (*SRD5A3*), and *pro-apoptotic WT1 regulator (PAWR*), may be involved in the formation of intersex traits. Furthermore, the copy number variations of the four regions, chr9: 71660801–71662800, chr1: 50776001–50778000, chr4: 58119201–58121600, and chr16: 778801–780800, may affect the expression of the *zinc finger protein*, *FOG family member 2* (*ZFPM2*), *LIM homeobox 8* (*LHX8*), *inner mitochondrial membrane peptidase subunit 2* (*IMMP2L*) and *slit guidance ligand 3* (*SLIT3*) genes, respectively, which contribute to the appearance of intersex traits. These results may supply a theoretical basis for the timely detection and elimination of intersex individuals in sheep, which could accelerate the healthy development of animal husbandry.

## 1. Introduction

Intersexuality, also known as hermaphroditism, refers to the phenomenon that a dioecious animal is characterized by female-to-male sex reversal or abnormal gonad development. Intersex individuals are unable to reproduce, which poses certain obstacles to the protection and breeding of endangered species, and causes production loss to animal husbandry. In vertebrates, the mechanisms of sex determination are mainly divided into two types, genetic sex determination and environmental sex determination [1]. Intersex mostly occurs in the goat population, with high occurrence frequencies (about 3%–10%) [2], while related reports on horses, donkeys, pigs, and sheep are few. In goats, it is also named polled intersex syndrome (PIS) for the phenomenon of intersex individuals often found in hornless goat populations [2]. In the previous study, a 11.7 kb deletion fragment containing a repeat sequence (AF404302) was cloned by PCR, and the complete absence of this fragment resulted in goat polled syndrome [3,4]. A recent study about long-read whole-genome sequencing of a PIS-affected goat and a horned control goat revealed the presence of a more complex structural variant consisting of a 10,159 bp deletion and an inversely inserted 480 kb duplicated segment containing two genes, *potassium inwardly rectifying channel subfamily J member 15* (*KCNJ15*) and *ETS transcription factor ERG* (*ERG*) [5]. The deletion of the PIS region was identified affecting the development of germ cell support cells, and it can also affect the expression of genes, including *Forkhead box L2* (*FOXL2*), *PIS-regulated transcript 1* (*PISRT1*), and *promoter FOXL2 inverse complementary* (*PFOXic*) [6], indicating that the lack of the PIS region is closely related to goat intersex traits.

Compared to goats, reports of intersex sheep are quite rare. Domestic sheep and domestic goats, diverging about 4 to 5 million years ago and evolving into two different branches, are relatively close in genetic distance, and they have many similar genetic targets during domestication [7]. Therefore, it was suspected that the cause of sexual traits in sheep may be similar to that of goat sex. The location of homologous sequences of goat PIS regions should be detected in sheep.

Genetic variations or regulatory regions may affect an individual’s phenotypic traits by affecting the transcription or translation of key genes [8]. In bovines, a 110 kb deletion in the *MER1 repeat containing imprinted transcript 1* (*MIMT1*) gene was a prominent cause of bovine abortion and stillbirth [9]. Bovine osteosclerosis may be associated with a deletion of approximately 2.8 kb in exon 2 and part of exon 3 of the *solute carrier family 4 member 2* (*SLC4A2*) gene encoding an anion exchanger [10]. An increased copy number of the *prolactin receptor* (*PRLR*) and *sperm flagellar 2* (*SPEF2*) genes in the K locus on the Z chromosome in chickens were closely related to the slow feathering trait of the chicken [11,12]. Additionally, the copy number variation of the *sperm flagellar 2* (*ASIP*) gene in sheep was closely related to the coat color [13]. It was speculated that some mutations may lead to the abnormal expression of certain genes that affect sex formation, leading to the generation of intersex traits.

As an excellent sheep variety of meat and wool, Lanzhou fat-tailed (LFT) sheep are famous for their large and fat tail and a number of excellent characteristics, such as the crude feed tolerance, higher disease resistance, and higher resilience than other domestic sheep. In recent years, only two surviving intersexual sheep were found in the LFT sheep population. In this study, the genome-wide resequencing of two intersex LFT sheep and two normal LFT sheep was performed, and combined with the resequencing data of four normal Tan sheep to find potential genes or regions related with the formation of ovine intersex traits, and thus provide an important basis for the timely detection and elimination of intersex individuals in sheep conservation and expansion.

## 2. Materials and Methods

### 2.1. Ethics Statement

All implemented experiments were approved by the Institutional Animal Care and Use Committee and were in strict accordance with good animal practices as defined by the Northwest A&F University (protocol number NWAFAC1008).

### 2.2. Animal and Sequencing

In this study, the ear tissues of four sheep, including two surviving intersex (LZ1 and LZ2) and six normal (LZ3 to LZ8) Lanzhou fat-tail sheep from Lanzhou City were collected and stored in 70% alcohol at −80 °C. Genomic DNA was extracted from sheep ear tissues using the phenol-chloroform method according to a previously reported protocol [14]. The DNA samples were quantified using a Nanodrop 1000 (Thermo Scientific, Waltham, MA, USA). Four DNA libraries, two intersex (LZ1 and LZ2) and two normal LFT sheep (LZ3 and LZ4), with insert sizes of approximately 350 bp, were constructed following the manufacturer’s instructions, and 150 bp paired-end reads were generated using the Illumina HiSeq X10 platform.

### 2.3. Sequence Quality Checking and Mapping

Considering the relatively close genetic relationship between LFT sheep and Tan sheep, the previous four resequencing data of Tan sheep were also used as a normal control group for further analysis [15,16]. Before alignment, the FastQC software was used (http://www.bioinformatics.babraham.ac.uk/projects/fastqc/) to detect the joint information, the length information, and the quality information for each base on each read of all the raw data. Based on the results of the above quality control, the adapter and low quality raw paired reads were filtered using Trimmomatic (v0.36) [17]. The high-quality reads were mapped to the sheep version 4.0 reference genome (GCF_000298735.2_Oar_v4.0_genomic.fna) using the ‘mem’ algorithm of the Burrows–Wheeler Alignment Tool (BWA) software [18]. SAMtools (http://samtools.sourceforge.net/) was used to remove replicate sequences [19].

### 2.4. Calling and Validation of SNPs and CNVs

The calling of single nucleotide polymorphisms (SNPs) was performed with Genome Analysis Toolkit (GATK, version 2.4–9) UnifiedGenotyper [20], and was annotated by ANNOVAR [21]. The called SNPs with reads > 4 and quality ≥ 20 were used for further analysis. CNVcaller software (https://github.com/JiangYuLab/CNVcaller) was used for the detection of whole-genome copy number variation (CNV) [22]. The specific steps were in accordance with a reported study [23], including segmenting the reference genome into a window of a specified size (800 bp) with a step size of 400 bp. Herein, Vst was used to measure the difference in the size of each copy number variation between different groups [24,25]. The mean log2 ratio across all probes falling within a specific CNV region was calculated. The variance of the means was for the entire set (Vt). The average variance within populations was then calculated (Vs) by taking the mean between populations (i.e., the V intersex sheep and Vnormal sheep). Vst values were finally calculated using the standard formula Vst = (Vt − Vs)/Vt.

### 2.5. Regional Localization and Depth Statistics for Ovine Homologous Sequences of PIS

The sequence of the PIS region was extracted from the goat reference genome (GCF_001704415.1_ASM170441v1_genomic.fna) by using SAMtools and then aligned to the sheep reference genome using BLAT v. 36 × 1 software (BLAST-Like Alignment Tool) to obtain the ovine homologous regions [26].

After that, we used the SAMtools-depth to count the reads depth for each locus in the candidate region. Furthermore, we corrected it based on the total depth of sequencing to obtain the reads depth in the homologous regions of the eight samples, including two intersex LFT sheep, two LFT sheep, and four reported Tan sheep [15,16]. If the reads depth was essentially 0, the candidate region was completely missing on the ovine genome.

### 2.6. PCR Amplification of PIS Candidate Region Sequences in Sheep

In order to further verify whether the PIS candidate region of the intersex individual was really missing, primers, namely PIS-1, PIS-2, PIS-3, and PIS-4, for the homologous sequence in the ovine PIS region were designed to reference the second generation genome of the sheep chr1: 247747059–247755846 sequence (Supplemental Table S1). We performed assays of amplification on two intersex and six normal LFT sheep according to previous reaction volume and amplification procedure [27]. The products were separated on 2.5% agarose gels.

### 2.7. Sweep Analysis of SNPs in Selected Regions

In order to identify the selection signatures in the genomes of sheep, two sequenced pools based on genetic differentiation (Fst) of each 150 KB genome window were separately performed. The specific formula of Fst was consistent with the previous studies [28,29].

After Z-transformation of Fst, the candidate selection windows were used by selecting the top 1% in Fst score intersections [30]. Finally, the geneview module of python and R were utilized for data visualization.

### 2.8. Gene Annotation and Functional Enrichment Analysis of Selected Signal Regions

Gene functional enrichment analysis was performed primarily on the KOBAS 3.0 website (http://kobas.cbi.pku.edu.cn/index.php) [31]. Herein, considering that the sheep database in KOBAS was not complete, a Perl script was used to compare the longest protein sequences of each gene in sheep and human by invoking Blastp, and the gene extracted in the previous step (2.4 and 2.7) was converted into a gene homologous to "human", and then human was selected as a species for annotation.

## 3. Results

### 3.1. Sequencing, Filtering, and Mapping

Whole-genome sequencing of two normal Tan sheep, as well as two intersex sheep and four normal LFT sheep, was performed on an Illumina HiSeq X10 platform using genomic DNA and 198.5 Gb of high quality paired-end reads were generated. After getting the raw data, we utilized FastQC software (http://www.bioinformatics.babraham.ac.uk/projects/fastqc/) for quality testing. According to the test results, the clean reads were obtained using trimmomatic (http://www.usadellab.org/cms/index.php/page=trimmomatic) to filter the low-quality reads (Supplemental Table S2). The clean reads of eight sheep were mapped to the sheep reference genome (GCF_000298735.2_Oar_v4.0_genomic.fna) using BWA-MEM [17,31], with an average mapping rate above 94% (Supplemental Table S3).

### 3.2. Single Nucleotide Polymorphisms (SNPs) Calling and Annotation

**Figure 1:**
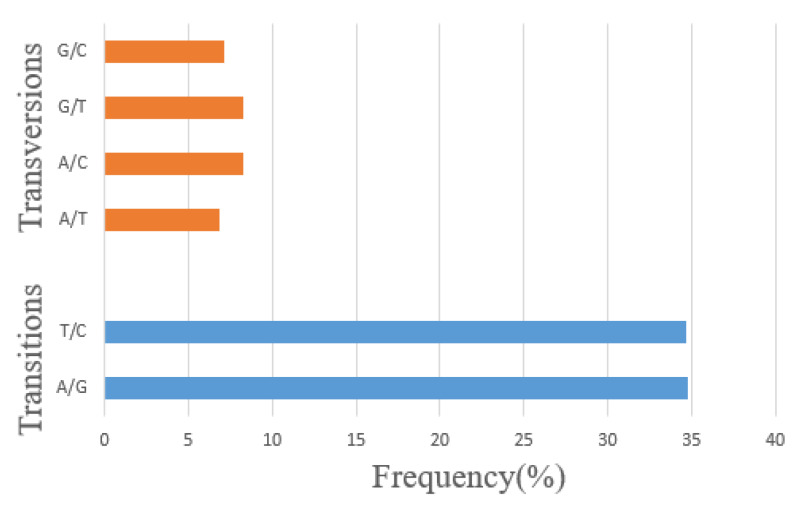
The statistics of mutation type of single nucleotide polymorphisms (SNPs). Note: The proportion of translation (ts) mutations and transversion (tv) mutations meet the 3 to 1 ratio.

A total of 29,732,629 SNPs were obtained among eight sheep, among which, the most distributed SNPs were on chromosome 1 (n = 3,186,739), and the least distributed, lowest number of SNPs were on chromosome 24 (n = 459,707) (Supplemental Table S4). According to the results of the annotation, the proportions of transition (ts) mutations (A/G 10333679 and T/C 10308562) and transversion (tv) mutations (A/T 2050141, A/C 2457813, G/T 2450228, and G/C 2132206) were 69.4% and 30.6%, respectively, which met the 3 to 1 ratio (Figure 1).

### 3.3. The Localization Results of Homologous Sequences of Goat PIS Region in Sheep

According to the specific information of the goat PIS region (AF404302.1: 27015–38775) in the NCBI, the obtained sequence of this 11.76 kb segment [2] was aligned to the sheep genome using BLAT, and then the score was calculated using the pslScore.pl script provided by the BLAT software to evaluate the results of the comparison.

**Figure 2:**
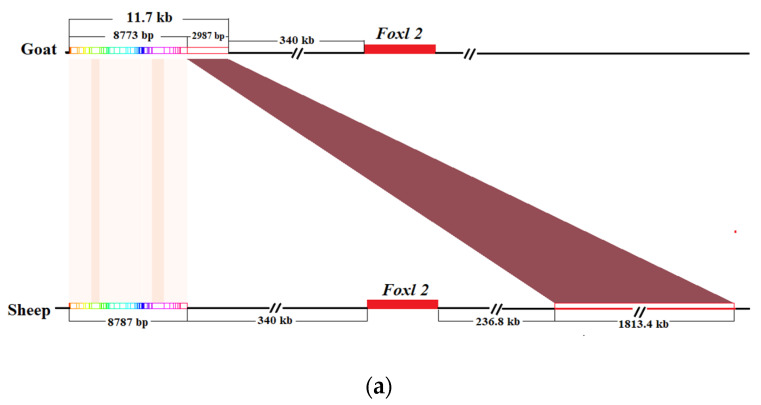

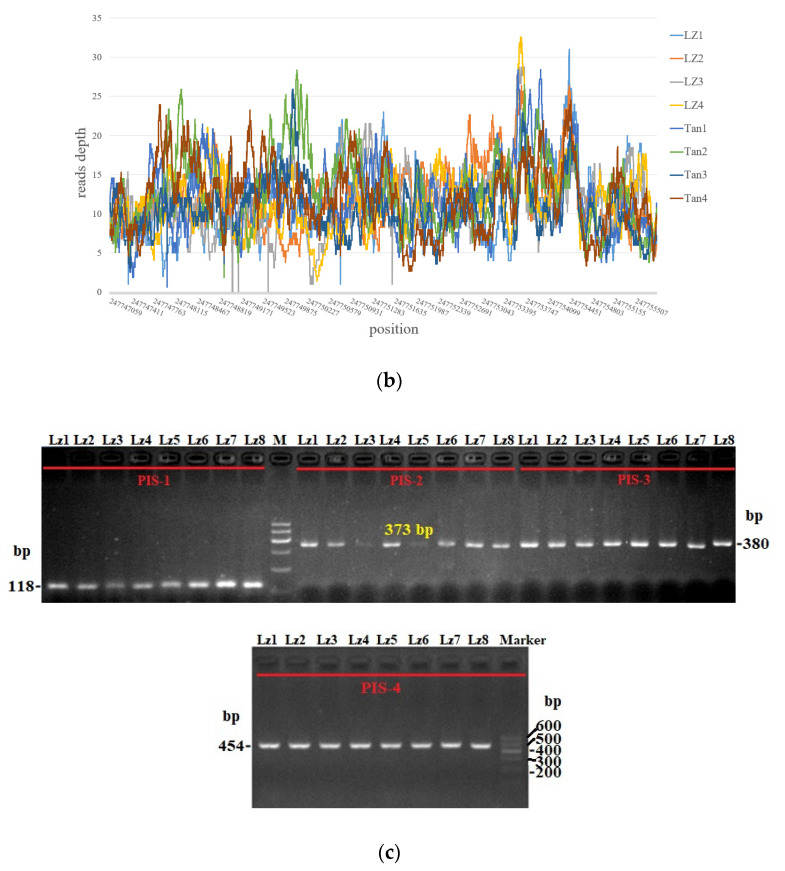
Study on the homologous sequences of the polled intersex syndrome (PIS) regions in sheep and goats. (**a**) The comparison results between the sheep chromosome 1: 247747059–250146105 and goat PIS area (the color line represents the matching relationship: goat 8773 bp matched sheep 8787 bp, and goat 2987 kb matched sheep 1813.4 kb). (**b**) The statistics of the read depth in the sheep 8787 bp candidate area. (**c**) Electrophoresis results of the four pairs of primer amplification products, indicating that the four regions of intersex sheep were not missing.

The BLAT results show that there were 79 alignment regions, and the alignment region with the highest score was located in the region of the ovine chromosome1 (start to end position: 247747059–250146105, 2399.046 kb) (Supplemental Table S5). Then, the detailed analysis of the 79 region revealed that the first 58 of them were a closely connected 8787 bp region (chr1: 247747059–247755846), corresponding to a segment of the 11.7 kb region of the goat (AF404302.1:30003–38775) with 100% coverage (Figure 2a). Further localization revealed that this region was located approximately 340 kb upstream of the *FOXL2* gene (chr1: 248088730–248095868) in the sheep genome, which was consistent with the position of the goat *FOXL2* gene. Therefore, the 8787 bp sequence (chr1: 247747059–247755846) was served as a candidate region for ovine intersex traits.

### 3.4. Statistics of Reads Depth and PCR Amplification of Sheep 8787 bp Candidate Sequences

In the two intersex sheep and normal individuals, the read depth was between 5 and 20, and there was no significant difference between them (Figure 2b).

According to the results of agarose gel electrophoresis, in two intersex LFT sheep (LZ1 and LZ2) and six normal LFT sheep (LZ3, LZ4, LZ5, LZ6, LZ7, and LZ8), four pairs of primers (PIS-1, PIS-2, PIS-3, and PIS-4) were able to amplify the target band in the candidate region, and the product lengths were 118, 376, 380, and 454 bp (Figure 2c), respectively, indicating that the candidate region of intersex sheep was not missing. It is further explained that the intersex traits of LFT sheep was not caused by the lack of the region.

### 3.5. Genome-Wide Selection Sweeping Analysis in Intersex and Normal Populations

**Figure 3:**
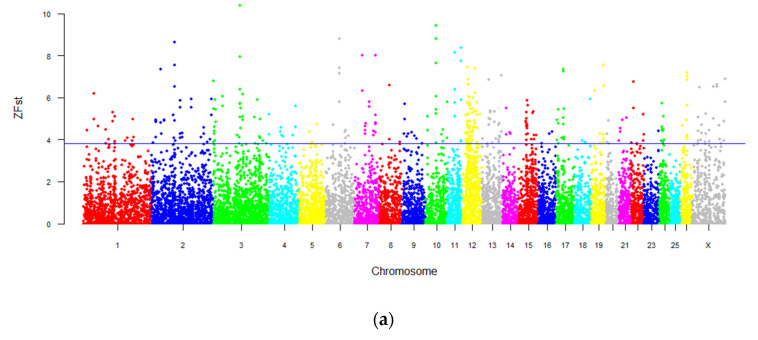

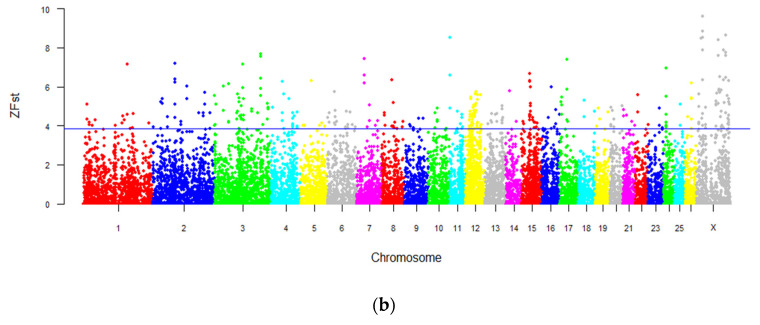
The detection of genome-wide selection signals in intersex and normal populations. (**a**) The genome-wide distribution of *ZFst* between intersex sheep and normal sheep (including normal Lanzhou large-tailed sheep and Tan sheep). (**b**) The genome-wide distribution of *ZFst* between intersex and normal individuals of Lanzhou large-tailed sheep. Note: The blue line represents the top 1% of the ZFst value.

*ZFst* score were calculated for the only two intersex LFT sheep and six normal sheep populations (including two LFT sheep and four Tan sheep). The top 1% percent of the windows with the highest *ZFst* score were defined as candidate selective sweep regions. The results show that most chromosomes contained windows with a higher differentiation coefficient, and chromosomes 2, 6, 7, 10, and 11 were strongly selected (Figure 3a). The region with the largest *ZFst* value was located on the X chromosome (chrX: 99675001–99825000, *ZFst* = 10.69), and the second one was located on chromosome 3 (chr3: 105000001–105150000, *ZFst* = 10.38) (Figure 3a; Supplemental Table S6).

A total of 466 genes were obtained by gene annotation (Supplemental Table S7), and then those genes were functionally enriched by KOBAS3.0. A total of 1757 significant entries were found (*p* < 0.05), and 1038 significant entries remained after the false discovery rate (FDR)-correction (corrected *p*-Value < 0.05), mainly including items, such as muscle development, fat development, and immunity. Furthermore, the pathways were screened and two more significant entries related to female gonadal development (GO: 0008585) and the development of primary female sexual characteristics (GO: 0046545) were found (*p* < 0.05) (Supplemental Table S7). Nevertheless, neither entry was significant after correction.

### 3.6. Genome-Wide Selection Sweeping Analysis in Intersex LFT Sheep and Normal LFT Sheep

To exclude the effects of SNP locus frequencies between different breeds, the study then compared two intersex individuals and two normal LFT sheep. Consistent with the previous section, the region with the largest *ZFst* value was located on the X chromosome (Figure 3b). By contrast, the second region was also in the X chromosome, rather than chromosome 3 (Figure 3b). Moreover, a total of 451 genes were obtained by annotation (Supplemental Table S8). After functional enrichment analysis, 1680 significant entries were found (*p* < 0.05) and 969 significant terms remained after false discovery rate (FDR)-correction (corrected *p*-value < 0.05) (Supplemental Table S9). Screening of the pathway revealed five significant entries related to the synthesis and response of the sex hormone (*p* < 0.05). After correction (corrected *p*-value < 0.05), the genes, such as *SRD5A2*, *SRD5A3*, and *PAWR*, were significantly rich in the androgen biosynthesis process and their responses to follicle stimulating hormones (Supplemental Table S9). Additionally, the genes involved in androgen receptor signaling pathways, androgen metabolism, and the regulation of intracellular estrogen receptor signaling pathways, and gonadotropin response processes, such as *UFM1 specific ligase 1* (*UFL1*), *GTP-binding nuclear protein Ran-like* (*LOC105609617*), *mediator complex subunit 14* (*MED14*), *DEAD-box helicase 5* (*DDX5*), etc. (Supplemental Table S9).

### 3.7. Detection of Genome-Wide Copy Number Variation (CNV) in Intersex and Normal Populations

As copy number variation regions (CNVRs) can be separated by gaps or poorly assembled regions, the adjacent initial calls were merged if their reads depth were highly correlated. The default parameters were as follows: the distance between the two initial calls was less than 20% of their combined length, and the Pearson’s correlation index of the two CNVRs was significant at the *p* = 0.01 level [21]. After calculation and combination through the CNVcaller software, 87,729 CNVRs were obtained, of which 1817 CNVRs were located on the scaffold sequence (not assembled into chromosomes) and 11,170 CNVRs were located on the X chromosome (Supplemental Table S10).

As the number of X chromosomes is different in females and males, the copy number variation on the scaffold and X chromosomes was not considered in the results. Therefore, 74,302 CNVRs located on autosomes were used for the subsequent analysis. As shown in Supplemental Table S10, the smallest proportion of CNVRs was chromosome 26 (5.8%), and the largest was chromosome 11 (9.92%). In addition, the number of CNVRs distributed on chromosome 1 (*n* = 8267) was the highest with a total length of 19,584,800 bp, while the smallest was on chromosome 26 (*n* = 1116) with 2,556,800 bp. The longest CNVR was 380,800 bp and located on chromosome 13 (Supplemental Table S10).

The copy number of the normal and intersex populations was screened with variation length > 2000 bp and Vst value > 0.25, and a total of 238 candidate regions were obtained. Gene annotation of the above 238 candidate CNVRs revealed that 140 were located in the intergenic region, and the remaining 98 overlapped with the genes (Supplemental Table S11). Functional enrichment analysis was performed on the annotated genes, revealing a total of 1838 significant entries (*p* < 0.05) and 1039 significant entries after FDR-correction (corrected *p*-value < 0.05) (Supplemental Table S12).

Through further GO enrichment analysis of CNVRs, four GO items related to female gonad development were found. Based on functional enrichment analysis, the enriched genes were mainly *ZFPM2, LHX8* (located at 43,507 nt downstream of its corresponding copy number region), IMMP2L (located at 400835 nt upstream of its corresponding copy number region), and *SLIT3* (Supplemental Table S12). The above four genes corresponded to four CNVRs, respectively. Furthermore, in the intersex individuals, the copy number region associated with the *LHX8*, *IMMP2L*, and *ZFPM2* genes were gained, while the copy number region associated with the *SLIT3* gene was lost (Table 1).

## 4. Discussion

It is well-known that the mutation of the goat PIS region contributes to the absence of horns and sex-reversal [4]. Herein, through the reads depth and the PCR amplification experiments, the PIS homologous segments of two intersex sheep and normal sheep were not in a missing state, which indicated that the intersex trait of LFT sheep may not be caused by the absence of this region. The intersexuality of goats was always accompanied by hornlessness, manifested as non-interval syndrome, and the probability of the intersex appearance in the hornless goat population was 3% to 10% [32].

Genetic variations or regulatory regions may affect an individual’s phenotypic traits by affecting the transcription or transition of key genes. We speculated that some mutations may lead to the abnormal expression of certain genes that affect sex formation, leading to the appearance of intersex. Furthermore, a previous study reported that the replication of the *SRY-box transcription factor 3* (*SOX3*) gene may also cause human developmental disorders [33]. An increased copy number of the *SRY-box transcription factor 9* (*SOX9*) gene may trigger the probability of intersex individuals [34], so it is suspected that the intersexuality of the sheep may be due to other reasons, such as genetic variations.

Functional enrichment analysis revealed significant entries for the androgen biosynthesis processes and follicle stimulating hormone responses, including *SRD5A2*, *SRD5A3*, and *PAWR* genes. Among them, mutations of the *SRD5A2*, which were closely related to testicular decline, could affect the formation of the urethra and external genitalia, leading to hypoplasia of the male reproductive organs [35]. Therefore, polymorphisms of the *SRD5A2* gene may be a key point leading to intersexuality in sheep.

Functional enrichment of the sequencing data for intersex sheep and normal sheep populations (including Tan sheep) did not find significant entries related to gonadal development or sex hormone metabolisms. A previous study reported that testicular tissue dysplasia was a key cause of human gender developmental disorders [35]. Additionally, the excessive synthesis of estrogen in vivo also affected the formation and development of female reproductive organs. Therefore, it was speculated that various mutations of genes related to the synthesis and secretion of androgen led to the fact that the testis and its accessory reproductive organs could not be maintained.

As hermaphroditism is not conducive to animal reproduction, it is speculated that intersexual individuals are selected to be eliminated in evolution, so the occurrence in the current group is low. A major limitation of this study is that there were only two intersexual individuals sequenced, which may result in a certain number of false positives in the sequencing results. If the limitation of the sample size is eliminated, the study may obtain more mutation regions or copy number variation regions that are more reliable than in this paper, and may even lock in the major genes that lead to intersex traits. It is undeniable that this study provides a direction and reference basis for further in-depth exploration of the molecular mechanism of sheep intersex traits.

## 5. Conclusions

This study was the first to locate the homologous sequence of the goat PIS region in sheep and found that the intersex traits of LFT sheep were not caused by the lack of this region. Through detecting the selective sweep regions, the vital genes associated with androgen biosynthesis and the follicle stimulating hormone response entry were found, including *SRD5A2*, *SRD5A3*, and *PAWR*. Additionally, the copy number variations of the four regions on chr9, chr1, chr4, and chr16 may affect the expression of the gonadal development genes, *ZFPM2*, *LHX8*, *IMMP2L*, and *SLIT3*, respectively, which contribute to the appearance of intersex traits.

## Figures and Tables

**Table 1:** The information of the four candidates copy number variation region and the genotype of eight samples.

CNV Regions			Related Genes	*V*st-Values	Sample Genotype							
Chromosome	Start Position	End Position			LZ1	LZ2	LZ3	LZ4	Tan1	Tan2	Tan3	Tan4
1	50776001	50778000	*LHX8*	0.5713	AB	AB	AA	AA	AA	AA	AA	AA
4	58119201	58121600	*IMMP2L*	0.7097	AB	AB	AA	AA	AA	AA	AA	AA
9	71660801	71662800	*ZFPM2*	0.8611	AB	AB	AA	AA	AA	AA	AA	AA
16	778801	780800	*SLIT3*	0.4357	Ad	Ad	AA	AA	AA	AA	AA	AA

Note: AA means two copies; AB means three copies; Ad means just one copy. *LHX8*, *LIM homeobox 8* gene; *IMMP2L*, *inner mitochondrial membrane peptidase subunit 2* gene; *ZFPM2*, *zinc finger protein FOG family member 2* gene; *SLIT3*, *slit guidance ligand 3* gene. The *V*st-values measure the difference in the size of each copy number variation between different groups.

## Supplementary Materials

The following are available online at https://www.mdpi.com/2076-2615/10/6/944/s1, Table S1: PCR amplification primer information for candidate region on sheep chromosome 1; Table S2: The statistics of resequencing samples from 4 Lanzhou large-tailed sheep; Table S3: The statistics of resequencing samples from 8 sheep; Table S4: Chromosome distribution of SNPs in sheep; Table S5: Goat PIS regional positioning on sheep genome (the top 10 score); Table S6: List of genes in the selected overlapping regions by top 1% highest Z(Fst) between intersex sheep and normal sheep (including normal Lanzhou large-tailed sheep and Tan sheep); Table S7: GO term of the candidate genes associated with the intersex; Table S8: List of genes in the selected overlapping regions by top 1% highest Z(Fst) between intersex and normal individuals of Lanzhou large-tailed sheep; Table S9: GO term of the candidate genes associated with the intersex; Table S10: Autosomal distribution of CNVRs in sheep; Table S11: Gene annotation of the candidate CNVRs; Table S12: Functional enrichment analysis of annotated genes.

## References

[1] Marshall Graves J.A. (2008). Weird animal genomes and the evolution of vertebrate sex and sex chromosomes. Annu. Rev. Genet..

[2] Eaton O.N. (1945). The relation between polled and hermaphroditic characters in dairy goats. Genetics.

[3] Pailhoux E., Vigier B., Chaffaux S. (2001). A 11.7-kb deletion triggers intersexuality and polledness in goats. Nat. Genet..

[4] Pailhoux E., Vigier B., Schibler L. (2005). Positional cloning of the PIS mutation in goats and its impact on understanding mammalian sex-differentiation. Genet. Sel. Evol..

[5] Simon R., Lischer H.E.L., Pieńkowska-Schelling A., Keller I., Häfliger I.M., Letko A., Schelling C., Lühken G., Drögemüller C. (2020). New genomic features of the polled intersex syndrome variant in goats unraveled by long-read whole-genome sequencing. Anim. Genet..

[6] Pannetier M., Renault L., Jolivet G. (2005). Ovarian-specific expression of a new gene regulated by the goat PIS region and transcribed by a FOXL2 bidirectional promoter. Gnomics.

[7] Alberto F.J., Boyer F., Orozco-terWengel P., Streeter I., Servin B., de Villemereuil P., Benjelloun B., Librado P., Biscarini F., Colli L. (2018). Convergent genomic signatures of domestication in sheep and goats. Nat. Commun..

[8] Wong K.K., de Leeuw R.J., Dosanjh N.S., Kimm L.R., Cheng Z., Horsman D.E., MacAulay C., Ng R.T., Brown C.J., Eichler E.E. (2007). A comprehensive analysis of common copy-number variations in the human genome. Am. J. Hum. Genet..

[9] Flisikowski K., Venhoranta H., Nowacka-Woszuk J. (2010). A novel mutation in the maternally imprinted PEG3 domain results in a loss of MIMT1 expression and causes abortions and stillbirths in cattle (Bos Taurus). PLoS ONE.

[10] Meyers S.N., McDaneld T.G., Swist S.L. (2010). A deletion mutation in bovine SLC4A2 is associated with osteopetrosis in Red Angus cattle. BMC Genom..

[11] Elferink M.G., Vallée A., Jungerius A.P. (2008). Partial duplication of the PRLR and SPEF2 genes at the late feathering locus in chicken. BMC Genom..

[12] Bu G., Huang G., Fu H. (2013). Characterization of the novel duplicated PRLR gene at the late-feathering K locus in Lohmann chickens. J. Mol. Endocrinol..

[13] Norris B.J., Whan V.A. (2008). A gene duplication affecting expression of the ovine ASIP gene is responsible for white and black sheep. Genome Res..

[14] Zhang S.H., Sun K., Bian Y.N., Zhao Q., Wang Z., Ji C.N., Li C. (2015). Developmental validation of an X-Insertion/Deletion polymorphism panel and application in HAN population of China. Sci. Rep..

[15] Wang X., Liu J., Niu Y., Li Y., Zhou S., Li C., Ma B., Kou Q., Petersen B., Sonstegard T. (2018). Low incidence of SNVs and indels in trio genomes of Cas9-mediated multiplex edited sheep. BMC Genom..

[16] Zhou S., Cai B., He C., Wang Y., Ding Q., Liu J., Liu Y., Ding Y., Zhao X., Li G. (2019). Programmable base editing of the sheep genome revealed no genome-wide off-target mutations. Front. Genet..

[17] Bolger A.M., Lohse M., Usadel B. (2014). Trimmomatic: A flexible trimmer for Illumina sequence data. Bioinformatics.

[18] Jiang Y., Xie M., Chen W., Talbot R., Maddox J.F., Faraut T., Wu C., Muzny D.M., Li Y., Zhang W. (2014). The sheep genome illuminates biology of the rumen and lipid metabolism. Science.

[19] Li H., Durbin R. (2009). Fast and accurate short read alignment with Burrows-Wheeler transform. Bioinformatics.

[20] McKenna A., Hanna M., Banks E., Sivachenko A., Cibulskis K., Kernytsky A., Garimella K., Altshuler D., Gabriel S., Daly M. (2010). The Genome Analysis Toolkit: A MapReduce framework for analyzing next-generation DNA sequencing data. Genome Res..

[21] Wang K., Li M., Hakonarson H. (2010). ANNOVAR: Functional annotation of genetic variants from high-throughput sequencing data. Nucleic Acids Res..

[22] Wang X., Zheng Z., Cai Y., Chen T. (2017). CNVcaller: Highly efficient and widely applicable software for detecting copy number variations in large populations. Gigascience.

[23] Gao Y., Jiang J., Yang S., Hou Y., Liu G.E., Zhang S., Zhang Q., Sun D. (2017). CNV discovery for milk composition traits in dairy cattle using whole genome resequencing. BMC Genom..

[24] Redon R., Ishikawa S., Fitch K.R., Feuk L., Perry G.H., Andrews T.D., Fiegler H., Shapero M.H., Carson A.R., Chen W. (2006). Global variation in copy number in the human genome. Nature.

[25] Sudmant P.H., Mallick S., Nelson B.J., Hormozdiari F., Krumm N., Huddleston J., Coe B.P., Baker C., Nordenfelt S., Bamshad M. (2015). Global diversity, population stratification, and selection of human copy-number variation. Science.

[26] Kent W. (2002). BLAT—The BLAST-like alignment tool. Genome Res..

[27] Li J., Zhang S., Erdenee S., Sun X., Dang R., Huang Y., Lei C., Chen H., Xu H., Cai Y. (2018). Nucleotide variants in prion-related protein (testis-specific) gene (PRNT) and effects on Chinese and Mongolian sheep phenotypes. Prion.

[28] Lai F.N., Zhai H.L., Cheng M., Ma J.Y., Cheng S.F., Ge W., Zhang G.L., Wang J.J., Zhang R.Q., Wang X. (2016). Whole-genome scanning for the litter size trait associated genes and SNPs under selection in dairy goat (Capra hircus). Sci. Rep..

[29] Guo J.H., Tao H.X., Li P.F., Li L., Zhong T., Wang L.J., Ma J., Chen X., Song T., Zhang H. (2018). Whole-genome sequencing reveals selection signatures associated with important traits in six goat breeds. Sci. Rep..

[30] Zhou Y., Connor E.E., Wiggans G.R., Lu Y., Tempelman R.J., Schroeder S.G., Chen H., Liu G.E. (2018). Genome-wide copy number variant analysis reveals variants associated with 10 diverse production traits in Holstein cattle. BMC Genom..

[31] Xie C., Mao X., Huang J., Ding Y., Wu J., Dong S., Kong L., Gao G., Li C.Y., Wei L. (2011). KOBAS 2.0: A web server for annotation and identification of enriched pathways and diseases. Nucleic Acids Res..

[32] E G.X., Jin M.L., Zhao Y.J., Li X.L., Li L.H., Yang B.G., Duan X.H., Hunag Y.F. (2019). Genome-wide analysis of Chongqing native intersexual goats using next-generation sequencing. 3 Biotech.

[33] Moalem S., Babul-Hirji R., Stavropolous D.J., Wherrett D., Bägli D.J., Thomas P., Chitayat D. (2012). XX male sex reversal with genital abnormalities associated with a de novo SOX3 gene duplication. Am. J. Med Genet..

[34] Kropatsch R., Dekomien G., Akkad D.A., Gerding W.M., Petrasch-Parwez E., Young N.D., Altmüller J., Nürnberg P., Gasser R.B., Epplen J.T. (2013). SOX9 duplication linked to intersex in deer. PLoS ONE.

[35] Jia W., Zheng D., Zhang L., Li C., Zhang X., Wang F., Guan Q., Fang L., Zhao J., Xu C. (2018). Clinical and molecular characterization of 5α-reductase type 2 deficiency due to mutations (p.Q6X, p. R246Q) in SRD5A2 gene. Endocr. J..

